# Efficacy of an herbal formula Guixiong Yimu San in preventing retained placenta and improving reproductive performance in cows

**DOI:** 10.1038/s41598-024-63521-x

**Published:** 2024-06-03

**Authors:** Dongan Cui, Lei Wang, Ling Wang, Jiongjie He, Yuqiong Li, Zhounian Zhang, Shengyi Wang

**Affiliations:** 1 Key Laboratory of New Animal Drug Project, Gansu Province; Key Laboratory of Veterinary Pharmaceutical Development, Ministry of Agriculture and Rural Affairs, Lanzhou Institute of Husbandry and Pharmaceutical Sciences of Chinese Academy of Agriculture Sciences, No. 335, Jiangouyan Street, Qilihe District, Lanzhou, 730050 Gansu China; 2 Institute of Animal Science, Ningxia Academy of Agricultural and Forestry Sciences, Yinchuan, China; 3 Livestock Station of Jiuduntan Ecological Construction Command in Liangzhou District, Wuwei, China

**Keywords:** Guixiong Yimu San, Herbal remedy, Retained placenta, Puerperal metritis, Prophylactic strategy, Cow, Zoology, Health care

## Abstract

Retained placenta is a common health issue, and appropriate prevention strategies are effective in postpartum health management. This study aimed to evaluate whether early intervention using GYS can prevent retained placenta and puerperal metritis, as well as enhance reproductive outcomes in cows. Each bovine in the GYS group (n = 591) received a single prophylactic dose of GYS (0.5 g/kg body weight) orally within 2 h after parturition, while those in the control group (n = 598) received no intervention. GYS treatment was associated with a decreased incidence of retained placenta (4.6% vs. 12.0%, *P* < 0.01, OR = 0.335), a lower puerperal metritis risk (8.8% vs. 20.1%, *P* < 0.01, OR = 0.369), and a reduced need for additional therapeutic antibiotics (11.2% vs. 26.1%, *P* < 0.01, OR = 0.342). We observed increases in the first service conception rate (59.7% vs. 49.1%, *P* < 0.01) and conception rate within 305 days postpartum (93.2% vs. 85.5%, *P* < 0.01) in the GYS group than in the control group. A significant decrease was observed in the number of services per conception (1.8 ± 1.1 vs. 2.1 ± 1.4, *P* < 0.01) and the calving-to-conception interval (83.6 ± 39.6 vs. 96.6 ± 52.5 days, *P* < 0.01) between the two groups. Additionally, GYS treatment increased milk yield on days 7, 14, and 28 postpartum without affecting milk fat, milk protein, somatic cell count (SCC), or milk urea nitrogen (MUN) on days 7 and 28 postpartum. Accordingly, the GYS was effective and safe in preventing retained placenta and to improve reproductive performance in cows. Therefore, it could be a prophylactic intervention for superior postpartum fertility in cows.

## Introduction

Retained placenta may be a risk factor for metabolic diseases during the transition period, resulting in substantial financial losses in dairy cattle^1^. The economic impact of retained placenta varies, with losses ranging from $311.9 to $456.2, depending on factors, including parity and herd management practices^2,3^. These costs are due to reduced production, treatment expenses, reproductive disorders, and an increased risk of culling. Retained placenta is the most common postpartum disorder, which can cause metritis or endometritis and affect fertility^3,4^. Retained placenta rates in cattle range from 4 to 18% globally^3,5, 6^ and from 5.5 to 29.1% in Chinese cattle^7,8^.

Retained placenta is a common health issue, and effective prevention strategies are crucial in postpartum health management. Current recommendations to prevent retained placenta in dairy cows include increasing comfort, reducing stress during calving, ensuring proper nutrition^9^, and implementing complementary treatments, including ethnoveterinary practices^10^. Impaired immune function is implicated in retained placenta^11^. Prophylactic vitamin E and selenium supplementation in nutritionally deficient cows ameliorate this condition^12^. Uterotonic agents, including prostaglandins (PGs) and oxytocin, are commonly administered to manage retained placenta after delivery in dairy cows^9^. However, recent studies reported that PGF2α does not improve the resolution of retained placenta or enhance reproductive performance^13^, while the efficacy of oxytocin treatment is controversial. Previous studies reported that prophylactic administration of oxytocin during calving does not markedly prevent retained placenta in dairy cows^13,14^. Conversely, Mollo et al.^15^ reported that postpartum oxytocin treatment substantially reduced the incidence of retained placenta and enhanced fertility in cows. The efficacy of oxytocin depends on the specific calving conditions of the cow. Magata et al.^16^ reported that administering oxytocin between 3 and 6 h postpartum prevented retained placenta and improved reproductive performance. Therefore, developing alternative strategies for managing retained placenta in cows is essential. Chinese herbal medicines are commonly administered to enhance postpartum health in female livestock^17,18^.

According to traditional Chinese veterinary medicine (TCVM) theory, blood stasis is a key factor in the pathogenesis of postpartum uterine diseases^19^, and herbal agents with blood circulation-activating activities can treat uterine disease during the early postpartum period in China^20–23^. GYS, modified from *Sheng Hua Tang*, is a classical herbal formula utilized in the therm postpartum women's care to stimulate blood circulation, increase uterine contraction, alleviate abdominal pain, and expel lochia^24^. GYS consists of Leonurus artemisia (*Laur.*) S.Y. Hu F, Angelica sinensis (*Oliv.*) Diels (radix), Ligusticum chuanxiong Hort. (radix), *Carthamus tinctorius *L. (Safflower), *Cyperus rotundus *L. (Cyperaceae), and *Glycyrrhiza uralensis FISCH* (radix). These herbs, when combined, could increase uterine contractions, alleviate abdominal pain, and relieve blood stasis syndrome in postpartum cows. Our preliminary studies suggest that GYS could be a therapeutic alternative for retained placenta in dairy cows^25^. Randomized clinical trials (RCTs) are considered the gold standard for clinical research. Here, we aimed to evaluate the efficacy and safety of prophylactic administration of GYS during the early postpartum phase in preventing retained placenta and determine whether GYS enhances subsequent reproductive performance in cows.

## Materials and methods

### Drugs

GYS (LOT No.: 20190916) was obtained from Beijing Kangmu Biotechnology Co., Ltd. (China). It is an herbal powder with 80–100 mesh particle size and is composed of *Leonurus artemisia* (*Laur.*) S.Y. Hu F, 120.0 g; *Angelica sinensis* (*Oliv.*) Diels (radix), 40.0 g; *Ligusticum chuanxiong* Hort*.* (radix), 40.0 g; *Carthamus tinctorius *L. (Safflower), 30.0 g; *Cyperus rotundus *L. (Cyperaceae), 40.0 g; and *Glycyrrhiza uralensis FISCH* (radix), 30 g. The herbal powder was administered as a single dose to cows weighing 600 kg (0.5 g crude drug/kg BW). Furthermore, high-performance liquid chromatography analysis revealed that the concentration of stachydrine hydrochloride, an active compound of *Leonurus artemisia* (*Laur.*) in GYS, was approximately 2.382 mg/g.

### Animals and herds

The trial was conducted at a large dairy farm in northwestern China between October 2019 and May 2020. The cows were transitioned to open housing with straw bedding before calving and fed a daily total mixed ration based on lactation stage and milk yield. The cows were fed ad libitum with 5% feed refusal, had access to water, and were milked thrice daily with an average yield of 12,000–13,000 kg per lactation. After 45 days postpartum, cows were artificially inseminated (AI) upon identifying estrus. Pregnancy was diagnosed using transrectal palpation and ultrasonography between 36 and 42 days after the last AI event. A second examination was performed 25–28 days later to confirm the pregnancy.

### Enrollment criteria

We enrolled animals between the ages of 2 and 7 years with a body condition score (BCS) ranging from 2.75 to 4.0 during the peripartum stage^26^. These animals were registered with Dairy Herd Improvement's Determination Lab and had available DHI records, including milk yield, fat content, protein levels, somatic cell count (SCC), and milk urea nitrogen (MUN). Abortion was defined as the termination of a pregnancy at 7 months or later, with the abortion date recorded as the parturition date. Dystocia was classified using a 1 to 5 scale based on the farm management strategy, where 1 indicated no assistance, and 5 denoted a surgical procedure^27^.

### Exclusion criteria

Herein, cows with acute postpartum diarrhea, lameness, displaced abomasum, or dystocia requiring assistance levels 4 and 5 were excluded. Additionally, cows with incomplete treatment adherence, deviations from the treatment protocol, or withdrawals from the Dairy Herd Improvement (DHI) milk recording program during the study period were excluded.

### Study design and clinical examination schedules

**Figure 1:**
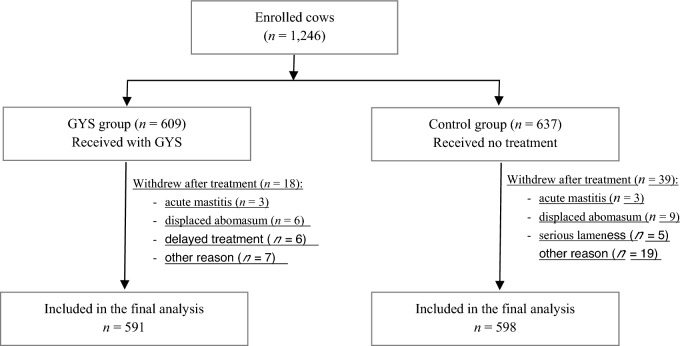
Flow diagram of the cow enrollment process, randomization of enrolled cows, reasons for excluding cows from analyses and cows included in the statistical analysis.

Herein, 1839 cows were calved, of which 1246 were enrolled and randomized into two groups using a three-digit number assigned sequentially. During the observation period, 57 cows were withdrawn from the trial based on exclusion criteria. Exclusion criteria for the GYS group include acute mastitis in three cows, displaced abomasum in two cows, failure to administer the herbal powder within 2 h post-calving due to competing tasks in six cows, and various other reasons that affected seven cows. Exclusion criteria for the control group included acute postpartum diarrhea in nine cows, displaced abomasum in three cows, acute mastitis in three cows, severe lameness in five cows, and various other reasons that affected five cows. We established two groups for further analysis: the GYS group (n = 591) and the control group (n = 598). Each group consisted of cows housed in separate pens. Figure 1 illustrates the disposition of the enrolled cows. No differences were observed in body condition score (BCS) at calving, parity, age, incidence of dystocia, or abortion rates between the herbal and control groups (Supplementary Tables S2, S3).

Herein, the retained placenta was diagnosed when fetal membranes were present at least 12 h postpartum. The diagnostic process included perineal examination, placental expulsion surveillance, and obstetric assessments, including uterine palpation through rectal examination or vaginoscopy, conducted 16 (± 4) h postpartum following the cleansing and disinfection of the perineal region. All enrolled cows were examined at 48 (± 6) h intervals within 21 days postpartum by a farm veterinarian; cows presenting with fetid, reddish-brown, watery vulvar discharge and a rectal temperature exceeding 39.5 °C were diagnosed with puerperal metritis following the protocol by Sheldon et al.^28^. In both groups, cows diagnosed with retained placenta or puerperal metritis were treated with ceftiofur (ceftiofur hydrochloride, Qilu Pharmaceutical Co., Ltd., Jinan, China) at a dose of 2.2 mg/kg body weight for 3 days. Both groups did not receive antibiotics or manual removal of fetal membranes.

All enrolled cows were monitored according to farm health protocols up to day 305 postpartum. Baseline data included birth dates, abortion incidences, dystocia, twin births, retained placenta, metritis, the date of the first estrus, AI dates, and the number of insemination attempts required to achieve pregnancy. AI was initiated in cows exhibiting estrus signs following the VWP. Cows were inseminated multiple times until 305 days postpartum without being culled for reproductive issues prior to this period. Reproductive performance metrics included the time to first AI, duration until pregnancy, first AI conception rate, overall conception rate (the number of pregnant cows divided by the total inseminations multiplied by 100), and inseminations per conception (Supplementary Table S3). Cows not pregnant by 305 days in milk (DIM) were classified as open, regardless of their continued presence in the herd or later conception during lactation.

Milk yield data for days 7, 14, 21, and 28 postpartum were collected using herd management software (AfiFarm™, version 5.3, Israel) to assess the safety of GYS as a prophylactic measure in cows (Supplementary Table S3). Milk protein, fat percentage, somatic cell count (SCC), and milk urea nitrogen (MUN) concentrations for enrolled cows on days 7 and 28 postpartum were obtained from DHI records at the DHI Determination Lab. Additionally, these parameters for a subset of enrolled cows were measured using MilkoScan™ 7 RM and Fossomatic™ 7/7 DC (CombiFoss™ 7, FOSS, Denmark).

### Treatment protocol

In the GYS group, cows were administered a prophylactic regimen consisting of 0.5 g/kg body weight (BW) of herbal powder orally within two hours postpartum via an esophageal tube directly into the rumen. The herbal powder was dissolved and vigorously stirred in 5.0 L of lukewarm water (36 ± 2 °C). The cows in the control group received no treatment.

### Efficacy measurements

The primary outcome measure evaluated the effect of GYS on the incidence of retained placenta and puerperal metritis in cows, utilizing the previously established diagnostic criteria. Secondary outcome measures focused on subsequent fertility in cows, including the proportion of first AI conceptions, the interval to first AI, the duration until pregnancy, the number of services per conception, and the proportion of cows conceiving within 305 days postpartum. Additionally, milk yield and parameters such as milk fat, milk protein, somatic cell count (SCC), and milk urea nitrogen (MUN) were assessed to evaluate the safety of GYS as a prophylactic intervention in cows.

### Statistical analysis

Means (SDs) were calculated to summarize continuous variables. The proportion of enrolled cows in each category was calculated for categorical variables. The Student’s *t*-test, the Mann–Whitney test, and the Chi-square test, as appropriate, were utilized to compare the days to first service, services until conception, and days to conception between the two groups. The proportions of cows with retained placenta, puerperal metritis, and those that required additional therapeutic antibiotics between the groups were modeled in a binary logistic regression model with dystocia and stillbirth as covariates. Additionally, the first AI conception and conception after all inseminations (total conception rate) were tested with a chi-squared test. The days open for cows that did conceive were determined using the Kaplan–Meier model of the survival analysis method, and the statistical differences in the observed survival curves of the two groups were analyzed using a log-rank test. To test the hypotheses in this study, the control group was chosen as the reference group for all logistic regression models and survival analyses. Statistical analyses were conducted using SPSS (version 25.0, IBM SPSS Statistics; New York, USA) and GraphPad Prism (GraphPad Software, Inc., CA 92037, USA) version 8.4. P < 0.05 was considered to be statistically significant.

### Ethical approval

The experimental procedures were meticulously followed, in accordance with the Institutional Guidelines for the Care and Use of Laboratory Animals and the ARRIVE guidelines. Approval for the animal study was granted by the Animal Care and Welfare Committee of the Lanzhou Institute of Husbandry and Pharmaceutical Sciences (Registration No. 2019-012).

## Results

### GYS lowered the risk of retained placenta and puerperal metritis

Based on visual inspection of the perineum and palpation of the uterus through the rectum and using vaginoscopy, 564 cows in the GYS group spontaneously expelled their placentae within 12 h after calving, while 27 cows were diagnosed with retained placenta; retained placenta occurred in 72 of 598 cows in the control group. In the GYS group, the odd ratio (OR) of the occurrence of retained placenta treated with GYS was 0.335 times (95% CI: 0.210–0.535, *P* < 0.01) compared to the control group. Puerperal metritis was diagnosed in 52 and 120 cows in the GYS and control groups, respectively. Furthermore, the number of cows that required additional therapeutic antibiotics was fewer in the GYS group than in the control group (11.2% vs. 26.1%, *P* < 0.01, OR = 0.342). The logistic regression analysis indicated that the OR between the two groups was 0.369 times (95% CI: 0.210–0.535, *P* < 0.01) and 0.342 times (95% CI: 0.248–0.471, *P* < 0.01).

### Herbal treatment improved subsequent reproductive performance in cows

**Figure 2:**
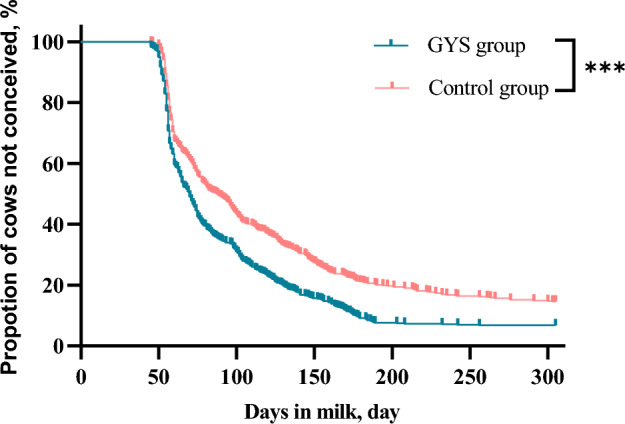
Survival curves for days to pregnancy in cows received one prophylactic dose (0.5 g/kg BW) of the herbal powder orally less than 2 h after delivery via an esophageal tube inserted directly into the rumen, or the controls receiving no intervention.

Table 1 depicts the effects of GYS treatment on reproductive performance. The first service conception rate (*P* < 0.01) and conception rate at 305 DIM (*P* < 0.01) were significantly higher in the GYS group than in the control group. Figure 2 illustrates that more cows were conceived in the GYS group than in the control group within 200 days postpartum (*P* < 0.001). The number of services per conception was significantly lower in the GYS group than in the control group (*P* < 0.01). The median number of days to conception was shorter in the GYS group than in the control group (*P* < 0.01). However, the difference between the two groups in the median number of days to first service was not statistically significant (*P* > 0.05).

**Table 1:** Effect of GYS on reproductive parameters in dairy cows.

Reproductive parameters	GYS group (*n* = 591)	Control group (*n* = 598)	*P*-value
Days to first service (day)	58.8 ± 8.0	59.0 ± 8.0	0.772
First service conception rate (%)	59.7	49.1	< 0.01
Services until conception	1.8 ± 1.1	2.1 ± 1.4	< 0.01
Days to conception (day)^1^	83.6 ± 39.6	96.6 ± 52.5	< 0.01
Conception rate at 305 DIM (%)^2^	93.2	85.5	< 0.01

*DIM* days in milk.

^1^Median number of days to conception was analyzed based on the cows that were pregnant within 305 days postpartum.

^2^Percentage of cows pregnant at 305 days postpartum expressed as a percentage of the inseminated animals in the respective group.

### Effect of GYS on milk yields and milk parameters in dairy cows

Tables 2 and 3 depict the effects of GYS treatment on milk yields and milk parameters. Milk yield at days 7, 14, and 28 postpartum was significantly higher in the GYS group than in the control group (*P* < 0.01), and no significant difference was observed in milk yield at day 21 between the two groups (*P* > 0.05). No significant differences were observed in milk fat, milk protein, SCC, and MUN concentration at days 7 and 28 postpartum (*P* > 0.05).

**Table 2:** Effect of GYS on milk yields at day 28 postpartum in dairy cows.

Milk yields (kg/d)	GYS group (*n* = 591)	Control group (*n* = 598)	*P*-value
Day 7	32.23 ± 10.46	29.29 ± 9.79	< 0.001
Day 14	36.96 ± 11.43	33.73 ± 10.37	< 0.001
Day 21	38.71 ± 12.01	38.08 ± 11.31	0.351
Day 28	41.18 ± 12.61	39.17 ± 11.91	0.005

**Table 3:** Effect of GYS on milk parameters in dairy cows.

Milk parameters postpartum		GYS group (*n* = 591)	Control group (*n* = 598)	*P*-value
Fat (%)	Day 7	5.03 ± 1.15	4.90 ± 1.19	0.059
	Day 28	4.68 ± 1.10	4.78 ± 1.02	0.101
Protein (%)	Day 7	3.57 ± 0.36	3.57 ± 0.43	0.765
	Day 28	3.33 ± 0.49	3.32 ± 0.34	0.663
SCC(× 10^3^/mL)	Day 7	139.31 ± 171.12	148.13 ± 169.29	0.372
	Day 28	120.62 ± 156.89	121.51 ± 168.78	0.925
MUN (mg/dL)	Day 7	13.09 ± 2.58	13.31 ± 2.47	0.133
	Day 28	12.89 ± 4.94	13.17 ± 2.81	0.076

*SCC* milk somatic cell count, *MUN* milk urea nitrogen.

## Discussion

The objective of this study was to investigate the effectiveness of early intervention using GYS in the prevention of retained placenta and puerperal metritis, as well as to assess the impact of GYS on the reproductive outcomes of cows. Retained placenta is a common uterine condition, and the adverse effects of retained placenta range from impaired reproductive performance to severe metritis with loss of production^9,29^. The working hypothesis is that oral GYS administration within 2 h postpartum accelerates expulsion of the placenta, lowers the risk of puerperal metritis, and enhances reproductive parameters, including the first-service conception rate, number of services per conception, median time to conception, and overall conception rate at 305 days in milk (DIM). Consequently, this study proposes an effective prophylactic strategy for preventing retained placenta in dairy cows.

According to TCVM theory, retained placenta is classified as blood stasis syndrome, a leading cause of postpartum diseases^19,24^. Herbal agents that activate blood circulation can treat uterine disease during the early postpartum period in China^20–23^. GYS regulates the balance between endothelin-1/nitric oxide (ET-1/NO) and thromboxane B_2_/6-keto-prostaglandin F_1α_ (TXB2/6-Keto-PGF_1α_), leading to blood stasis resolution in bovines with the retained placenta^25^. Herein, we observed a significant decrease in the incidence of retained placenta in the GYS group than in the control group for more than 12 h (OR = 0.335, *P* < 0.001). The incidence of postpartum uterine diseases is associated with immune function during the transition period^29^, with the immune response playing a pivotal role in the separation and expulsion of the placenta^30,31^. Th1 cells release proinflammatory cytokines, initiating an inflammatory response that induces apoptosis in trophoblast and endometrial epithelial cells, thereby facilitating placental detachment^31,32^. Huang et al.^25^ reported that GYS treatments could promote a Th1/Th2-type cytokine equilibrium by increasing IL-2 and TNF-α levels and decreasing those of IL-4 and IL-10. We demonstrated that GYS exhibited analgesic and anti-inflammatory effects, as evidenced by the outcomes of hot plate, writhing, ear edema, capillary permeability, and paw edema tests in mice (results not published). Additionally, a large dose of *Leonurus artemisia* (*Laur*.)* S.Y. Hu F* modulates uterine contractions and improves uterine hemorheology and microcirculation^33^. These effects might improve the uterine condition and overall postpartum physical health, contributing to a lower risk of retained placenta. GYS treatment reduced puerperal metritis risk in cows (OR = 0.369, *P* < 0.01). Herbal treatment may be beneficial in the uterine recovery process due to prompt placental detachment during the early postpartum stage. The number of cows that required therapeutic antibiotics was fewer in the GYS group than in the control group, consistent with the World Health Organization's guidelines for appropriate antibiotic use. These findings indicate that GYS may prevent postpartum uterine disease in dairy cows.

Expulsion of fetal membranes indicates successful parturition, potentially improving subsequent reproductive performance. Fertility is an essential characteristic of dairy cow production, and the postpartum period provides an opportune moment to initiate preventative measures that can assist cows in achieving optimal interpregnancy intervals. Herein, GYS treatment significantly reduced the duration of open periods in cows (83.6 ± 39.6 vs. 96.6 ± 52.5 days,* P* < 0.01) compared to the control group. The length of the open period is a critical measure of reproductive efficiency in cows. Huang et al.^25,34^ reported that GYS enhances uterine recovery and overall bovine health through mechanisms that improve blood circulation and possess anti-inflammatory, immunomodulatory, and antioxidative effects. Prophylactic administration of GYS improves the uterine environment by facilitating earlier placental detachment and reducing the risk of uterine infection, thereby fostering conditions conducive to higher conception rates. These aspects were evident in this study, in which we recorded a significant increase in the first service conception rate (OR = 0.578, *P* < 0.01) and conception rate at 305 DIM (OR = 0.424, *P* < 0.01) among cows in the GYS group than among those in the control group post-treatment. The conception rate serves as a comprehensive indicator of reproductive efficacy. The effectiveness of GYS was demonstrated by the higher conception rates within 200 days postpartum, as illustrated in the survival curves (Fig. 2). This indicates that early GYS intervention improves fertility in the early postpartum period, making it an effective prophylactic measure to boost reproductive performance in dairy cows.

Safe administration of herbal medicines and products is crucial in healthcare^35^. Our previous studies reported that oral administration of GYS is safe for mice and rats at doses < 90.0 g/kg body weight (BW) daily or 30.0 g/kg BW daily over 28 days^36^. Furthermore, cows in early lactation could tolerate oral doses of GYS up to 1.5 g/kg BW (threefold the recommended dosage) once daily for 3 days during target animal safety tests^37^. Herein, oral administration of GYS at a dose of 0.5 g/kg BW had no substantial effects on milk fat, milk protein, SCC, and MUN at days 7 and 28 postpartum. Variations in the parameters above can impact the quality of raw milk^38^. Therefore, these indices may serve as indicators to assess the safety of herbal medicines administered in treating lactating cows. We demonstrated that GYS did not affect milk composition, including milk fat, milk protein, SCC, or MUN. This finding suggested that GYS can enhance milk yield in early lactation cows because of the improved postpartum physical health following GYS administration. Thus, GYS is safe for postpartum cows, based on our findings.

## Conclusion

Administration of GYS as a prophylactic postpartum intervention is efficacious and safe in preventing retained placenta and to improve reproductive performance in cows. This strategy can save cost by reducing the number of inseminations, shortening the calving-to-conception interval, and lowering the risks of retained placenta and puerperal metritis without adverse effects. Our findings will provide guidelines for cattle postpartum fertility management.

### Supplementary Information

Supplementary Table S1.

Supplementary Table S2.

Supplementary Table S3.

## Data Availability

All data generated or analysed during this study are included in this published article and its Supplementary Table S3.

## Abbreviations

GYS: Guixiong Yimu San
SCC: Somatic cell count
MUN: Milk urea nitrogen
TCVM: Traditional Chinese veterinary medicine
RCTs: Randomized clinical trials
VWP: Voluntary waiting period
AI: Artificially inseminated
BCS: Body condition score
DIM: Days in milk
ET-1: Endothelin-1
NO: Nitric oxide
TXB_2_: Thromboxane B_2_
6-Keto-PGF_1α_: 6-Keto-prostaglandin F_1α_
Th1/Th2: (CIL-2 + CTNFa)/(CIL-4 + CIL10)
